# *Equisetum hyemale* L. Extracts: Phytochemistry, Biological Performance, ADMET Profiling, and Toxicity Predictions

**DOI:** 10.3390/ph18121901

**Published:** 2025-12-17

**Authors:** Yulianna Minutti-Calva, Karen Schürenkämper-Carrillo, Edwin E. Reza-Zaldívar, Oscar E. Del Razo-Rodríguez, Ian Vitola, Jorge Manuel Silva-Jara, José Daniel Lozada-Ramírez, Daniel A. Jacobo-Velázquez, Diego E. Navarro-López, Marco Chávez-Tinoco, Edgar R. López-Mena, Jorge L. Mejía-Méndez, Eugenio Sánchez-Arreola

**Affiliations:** 1 Departamento de Ciencias Químico-Biológicas, Universidad de las Américas Puebla, Ex Hacienda Sta. Catarina Mártir S/N, San Andrés Cholula 72810, Mexico; yulianna.minuttica@udlap.mx (Y.M.-C.); karen.schurenkamperco@udlap.mx (K.S.-C.); jose.lozada@udlap.mx (J.D.L.-R.); 2 Tecnologico de Monterrey, Escuela de Medicina y Ciencias de la Salud, Ave. Ignacio Morones Prieto 3000, Monterrey 64710, Mexico; edwin.reza@tec.mx; 3 Instituto de Ciencias Agropecuarias, Universidad Autónoma del Estado de Hidalgo, Santiago Tulantepec de Guerrero Lugo 43775, Mexico; oscare@uaeh.edu.mx; 4 Departamento de Farmacobiología, Centro Universitario de Ciencias Exactas e Ingenierías (CUCEI), Universidad de Guadalajara, Blvd. Marcelino García Barragán 1421, Olímpica, Guadalajara 44430, Mexico; ian.castro8800@alumnos.udg.mx (I.V.); jorge.silva@academicos.udg.mx (J.M.S.-J.); 5 Tecnologico de Monterrey, Escuela de Ingeniería y Ciencias, Av. Eugenio Garza Sada 2501 Sur, Monterrey 64849, Mexico; djacobov@tec.mx; 6 Tecnologico de Monterrey, Escuela de Ingeniería y Ciencias, Epigmenio González 500, San Pablo, Santiago de Querétaro 76130, Mexico; diegonl@tec.mx; 7 Departamento de Genética del Desarrollo y Fisiología Molecular, Instituto de Biotecnología (IBT), Universidad Nacional Autónoma de México (UNAM), Cuernavaca 62210, Mexico; marco.antonio.chavez@ibt.unam.mx; 8 Tecnologico de Monterrey, Escuela de Ingeniería y Ciencias, Av. Gral. Ramón Corona No 2514, Colonia Nuevo México, Zapopan 45121, Mexico

**Keywords:** traditional medicine, *Equisetum hyemale*, antibacterial, antioxidant, anticancer, nutritional value

## Abstract

**Background:** *Equisetum hyemale* L., commonly known as scouring rush or horsetail, is a perennial plant with significant applications in traditional medicine. **Methods:** The aerial parts of *E. hyemale* L. were macerated with hexane, chloroform, and ethyl acetate. The phytochemical profile of the extracts was investigated using chromatography approaches. The biological performance of the extracts was determined using antibacterial, antioxidant, anticancer, and toxicity in vitro and in vivo models. Molecular docking and ADMET analyses were employed to determine interactions with structural components of multidrug resistant bacteria and assess potential toxicological risks. **Results:** The extracts exert high scavenging activity against ABTS radicals (IC_50_ 2.57–2.68 μg/mL), but poor antibacterial activity. It was evidenced that treatment with extracts exerts in moderate cytotoxicity on hepatocellular and colorectal cancer cell lines. Toxicity assays unveiled that the extracts decrease the survival rate of *C. elegans* nematodes after 2 h of exposure to treatment. In silico studies evidenced a high affinity of campesterol and calcitriol towards the DNA gyrase, and the oral bioavailability of farnesol and limonene. **Conclusions:** Our findings demonstrated the presence of biologically active secondary metabolites in hexane, chloroform, and ethyl acetate extracts from *E. hyemale* L. This work also demonstrated the biological performance of these extracts in in vitro and in vivo models, and validated the potential pharmacokinetic and pharmacodynamic profile of their phytoconstituents.

## 1. Introduction

Traditional medicine (TM) is a fundamental resource in the prevention and treatment of infectious, metabolic, and neurological disorders. *Equisetaceae* is a monotypic family of herbaceous and perennial plants, characterized by their rhizomes and articulated stems and worldwide distribution [1]. The genus *Equisetum* comprises ~20 species, and it is divided into various subgenera (*Hippochaete*, *Distichium*, and *Mariscus*) with distinctive morphological features, specialized habitats, and capacity for adaptation to wetland environments. In TM, species from the genus *Equisetum*, such as *E. telmateia*, are used to treat kidney stones, stomach pain, and cystitis [2].

From the genus *Equisetum*, *E. hyemale* L., also known as horsetail, is a two-meter-tall species with brittle and cylindrical hollow stems, and dark green and spaced rings running around the stem [3,4]. Historically, preparations from *E. hyemale* L. have been considered for the treatment of acute stroke, hypertension, bleeding, and cancer [5]. In addition, it has been documented that extracts from *E. hyemale* L. can inhibit the growth of pathogenic bacteria involved in wound infections, enhance fibroblast collagen synthesis, promote the secretion of anti-inflammatory cytokines such as IL-10, and reduce tumor growth in immunodeficient BALB/c nude mice [6]. Despite the ample scientific evidence about the uses of *E. hyemale* L. in TM, few studies have scientifically validated non-polar and polar preparations of *E. hyemale* L. as potential alternatives for the treatment of disorders such as infections caused by high-priority pathogenic bacteria and cancer.

The current scientific evidence on the therapeutic applications of *E. hyemale* L. is mainly focused on using polar solvents such as methanol, ethanol, or water. However, it is important to note that non-polar solvents can also be leveraged to obtained extracts with wide biological performance due to the presence of terpenes, fatty acids, or steroids, which could be candidates for novel drug development [7]. From a chemical perspective, it is crucial to address that non-polar based preparations tend to dissolve in lipid environments, allowing them to penetrate biological membranes more readily than their polar counterparts [8]. From a biochemical perspective, the interaction between non-polar extracts and their targets often occurs through hydrophobic interactions, which can affect receptor binding and enzyme activity. Despite this, it is also noteworthy to mention that few studies have validated the toxicity of polar and non-polar preparations from *E. hyemale* L. either in vivo or in silico, which is imperative for identifying potential adverse effects and assessing their safety profiles to enable appropriate dosage determination, prolonged use, and regulatory compliance [9].

Therefore, considering the need to explore novel alternatives for bacterial infections, oxidative-related disorders, and cancer, this work aimed to obtain hexane, chloroform, and ethyl acetate extracts of the aerial parts of *E. hyemale* L. The phytochemical composition of the extracts was estimated using the TPC and TFC assays, GC/MS, and HPLC. The antibacterial activity of the obtained extracts was demonstrated against Gram-positive and Gram-negative strains. The anticancer activity of the extracts was analyzed using colon and hepatocellular cancer cell lines, as well as fibroblast and macrophage-derived cell lines. The antioxidant activity of the extracts was investigated through the DPPH, ABTS, and H_2_O_2_ assays. The toxicity of the extracts was evaluated utilizing *A. salina* nauplii and *C. elegans* in vivo models. Considering the data obtained from GC/MS, the main phytoconstituents were evaluated in silico for their molecular interaction with bacterial components and toxicity and pharmacokinetics profiles. The retrieved data from this study provides valuable insights into the potential therapeutic uses of *E. hyemale* L., highlighting its potential for antibacterial, anticancer, and antioxidant applications.

## 2. Results

### 2.1. Bromatological, TPC and TFC Analyses

The bromatological results from the aerial parts of *E. hyemale* L. are presented in Table S1. The TPC and TFC of the ethyl acetate extract were evaluated through calibration curves constructed with GAE and Qu. The TPC and TFC of ethyl acetate extract were 58.39 mg GAE/g and 18.71 mg Qu/g, respectively.

### 2.2. GC/MS and HPLC Analyses

The GC/MS analysis of the chemical composition of the hexane, chloroform, and ethyl acetate extracts of *E. hyemale* L. is presented in Table 1. The GC/MS chromatograms of each extract is presented in the Supplementary Materials as Figures S1, S2, and S3, respectively. An HPLC analysis was performed just for the ethyl acetate extract considering its polarity and the available literature (see Table 2). The chromatogram obtained from this analysis is presented in Figure S4.

### 2.3. Antibacterial Activity

The hexane, chloroform, and ethyl acetate extracts of *E. hyemale* L. were ineffective against the panel of Gram-positive (*Staphylococcus aureus*) and Gram-negative (*Escherichia coli*, *Klebsiella pneumoniae*, *Pseudomonas aeruginosa*, and *Proteus mirabilis*) strains used in this study at the concentrations of 5, 10, 50, and 100 mg/mL. The results are listed in Table S2.

### 2.4. Cytotoxicity Activity

**Figure 1:**
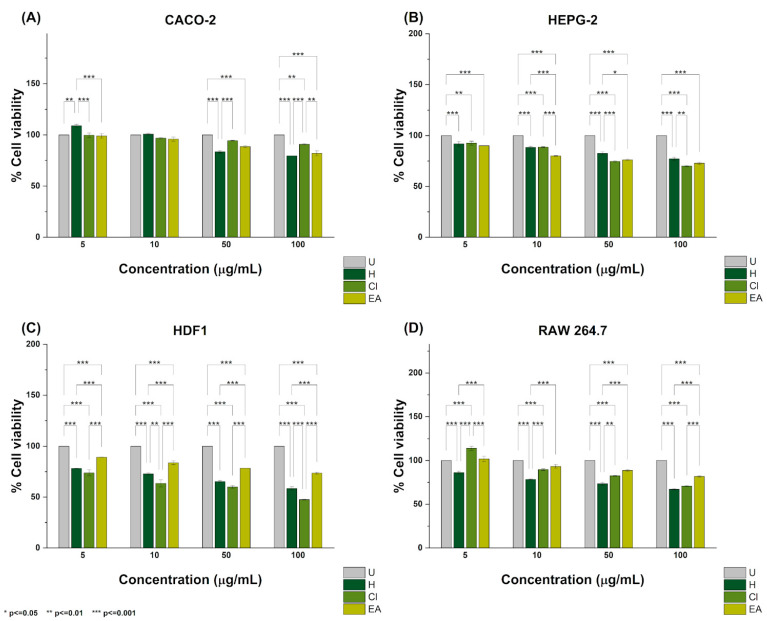
Cytotoxicity of the hexane, chloroform, and ethyl acetate extracts of *E. hyemale* L. against the (**A**) CACO-2, (**B**), HEPG-2, (**C**) HDF1, and (**D**) RAW 264.5 cell lines. * *p* < 0.05, ** *p* < 0.01, and *** *p* < 0.001. Samples consisted of hexane (H), chloroform (Cl), and ethyl acetate (EA) extracts. Untreated (U) cells were appraised as a negative control. Data are represented as the mean ± standard deviation (SD) from three independent experiments.

The cytotoxicity of the hexane, chloroform, and ethyl acetate extracts of *E. hyemale* L. against the CACO-2, HEPG-2, HDF-1, and RAW 264.7 lines is illustrated in Figure 1A. Against the CACO-2 cell line, treatment with 5 and 10 μg/mL of the hexane extract did not affect cell viability. However, at 50 and 100 μg/mL, it caused 16.44 and 20.50% of the cells to die, respectively. Conversely, treatment with 5, 10, and 50 μg/mL of the chloroform extract resulted in 0.33, 3.20, and 5.60% dead cells, respectively. At 100 μg/mL, it resulted in 9.19% dead cells. Similarly, treatment with 5 and 10 μg/mL of the ethyl acetate extract resulted in 1.01 and 4.07% dead cells. In contrast, treatment with 50 and 100 μg/mL resulted in 11.40 and 17.96% dead cells, respectively (see Figure 1A). Against the HEPG-2 cell line, it was noted that treatment with 5 and 10 μg/mL of the hexane extract caused the death of 8.12 and 11.60% of cells, whereas at 50 and 100 μg/mL, it resulted in 17.55 and 22.87% dead cells. Treatment with 5, 10, and 50 μg/mL of the chloroform extract resulted in 7.42%, 11.35%, and 25.45% cell death, respectively. At 100 μg/mL, it resulted in 29.94% dead cells. In comparison, treatment with 5, 10, and 50 μg/mL of the ethyl acetate extract resulted in 10.03%, 19.98%, and 23.86% dead cells, respectively. At 100 μg/mL, treatment with the ethyl acetate extract resulted in 27.22% dead cells (see Figure 1B).

Against the HDF-1 cell line, treatment with 5 and 10 μg/mL of the hexane extract resulted in the death of 21.88 and 27.17% of cells, whereas at 50 and 100 μg/mL, it induced the death of 34.78 and 41.56% of cells, respectively. Treatment with 5, 10, and 50 μg/mL of the chloroform extract resulted in the death of 26.21%, 36.61%, and 40.09% of the cells, respectively. Treatment with the chloroform extract at 100 μg/mL resulted in 52.54% dead cells. Similar to the hexane and chloroform extracts, treatment with 5 and 10 μg/mL of the ethyl acetate extract resulted in 10.89 and 16.33% dead cells, whereas at 50 and 100 μg/mL, it caused 21.62 and 26.50% of the HDF-1 cells to die, respectively (see Figure 1C).

As shown in the same figure, treatment with 5, 10, and 50 μg/mL of the hexane extract resulted in 13.82, 21.64, and 26.59% of the RAW 264.7 cells dying. At 100 μg/mL, the hexane extract caused the death of 32.85% of cells. Treatment with 5 μg/mL of the chloroform extract did not cause the death of RAW 264.7 cells; however, at 10, 50, and 100 μg/mL, the treatment resulted in 10.31, 17.47, and 29.26% cell death. Regarding the ethyl acetate extract, it was noted that treatment with 10, 50, and 100 μg/mL resulted in the death of 6.70, 11.34, and 18.22% of the cells. Like the chloroform extract, the ethyl acetate extract was not cytotoxic to the RAW 264.7 cell line at 5 μg/mL (see Figure 1D). Based on these findings, Table 3 lists the LC_50_ values of the hexane, chloroform, and ethyl acetate extracts against each cell line evaluated in this study.

### 2.5. Antioxidant Activity

**Figure 2:**
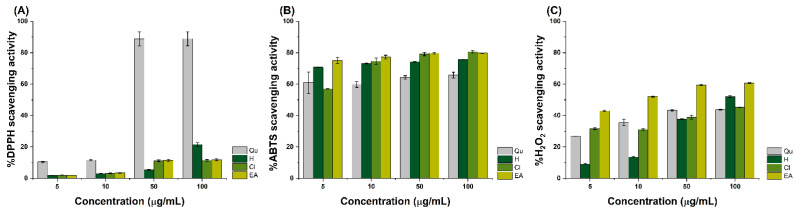
Antioxidant activity of the hexane, chloroform, and ethyl acetate extracts of *E. hyemale* L. against (**A**) DPPH, (**B**) ABTS, and (**C**) H_2_O_2_ radicals. Samples consisted of quercetin (Qu), hexane (H), chloroform (Cl), and ethyl acetate (EA) extracts. Data are represented as the mean ± standard deviation (SD) from three independent experiments.

The antioxidant activity of the hexane, chloroform, and ethyl acetate extracts of *E. hyemale* L. towards DPPH, ABTS, and H_2_O_2_ radicals were investigated. As shown in Figure 2A, treatment with the hexane extract at 5, and 10 µg/mL scavenged 1.85 and 2.96% of DPPH radicals, respectively. Moreover, at 50 and 100 µg/mL, the treatment resulted in the inhibition of 5.4 and 21.6% of the free radicals. In contrast, treatment with the chloroform extract at 5 and 10 µg/mL inhibited the formation of 1.97 and 3.21% of DPPH radicals. In contrast, at 50 and 100 µg/mL, it inhibited 11.25 and 11.38% of the free radicals, respectively. Treatment with 5 and 10 µg/mL of the ethyl acetate extract scavenged 1.95 and 3.48% of DPPH radicals. At 50 and 100 µg/mL, treatment with the ethyl acetate extract resulted in the scavenging of 11.54% and 11.9% of DPPH radicals.

As can be observed in Figure 2B, treatment with 5, 10, and 50 µg/mL of the hexane extract caused the inhibition of 70.81, 73.08, and 74.40% of ABTS radicals. At 100 μg/mL, treatment with hexane extract scavenged 75.61% of the free radicals. On the other hand, treatment with 5 and 10 μg/mL of the chloroform extract scavenged 57.01% and 74.6% of the radicals, whereas at 50 and 100 μg/mL, it inhibited the formation of 79.14% and 80.62% of the ABTS radicals, respectively. Treatment with the ethyl acetate extract at 5 and 10 μg/mL resulted in the inhibition of 75 and 77.21% of the free radicals. At 50 and 100 μg/mL, treatment with the ethyl acetate extract scavenged 79.73 and 79.78% of ABTS radicals, respectively. As noted in Figure 2C, treatment with 5 and 10 μg/mL of the hexane extract inhibited the formation of 9.04% and 28.25% of H_2_O_2_ radicals, whereas at 50 and 100 μg/mL, it scavenged 37.61% and 52.09% of the free radicals, respectively. Treatment with the chloroform extract at 5, 10, and 50 μg/mL scavenged 42.95, 51.99, and 59.42% of H_2_O_2_ radicals, respectively. At 100 μg/mL, treatment with the chloroform extract inhibited the formation of 60.64% of the free radicals. Similarly, treatment with 5 and 10 μg/mL of the ethyl acetate extract scavenged 31.65 and 31.13% of the free radicals, whereas at 50 and 100 μg/mL, it inhibited the generation of 38.92 and 45.31% of H_2_O_2_ radicals, respectively. The IC_50_ values of the extracts against DPPH, ABTS, and H_2_O_2_ are presented in Table 4.

### 2.6. Toxicity Analysis

**Figure 3:**
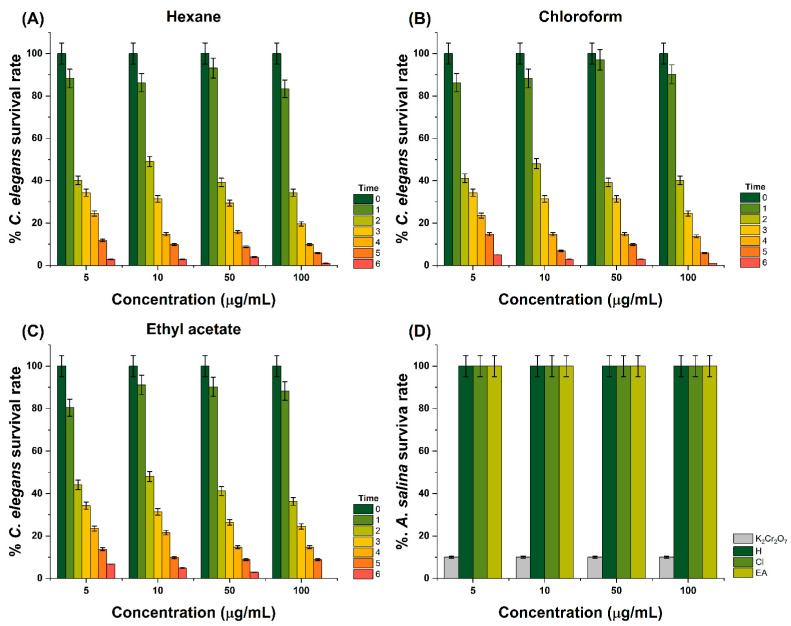
Toxicity of the hexane, chloroform, and ethyl acetate extracts of *E. hyemale* L. against the (**A**–**C**) *C. elegans* nematodes and (**D**) *A. salina* nauplii. Samples consisted of potassium dichromate (K_2_Cr_2_O_7_), hexane (H), chloroform (Cl), and ethyl acetate (EA) extracts. Data are represented as the mean ± standard deviation (SD) from three independent experiments.

The toxicity of the hexane, chloroform, and ethyl acetate extracts of *E. hyemale* L. were evaluated against *A. salina* nauplii and *C. elegans* nematodes. As represented in Figure 3A, treatment with the hexane extract decreased the viability of *C. elegans* nematodes in a dose-dependent manner. For instance, it was determined that treatment with 5–100 μg/mL resulted in 93.13–83.33% viability after 1 h of exposure to the treatment. A 6 h exposure to treatment with the hexane extract resulted in a 3.92–0.98% survival rate. In comparison, treatment with 5–100 μg/mL of the chloroform extract resulted in 97.05–86.27% and 2.94–0.98% survival rates after 1 and 6 h of exposure to the treatment, respectively. Regarding treatment with the ethyl acetate extract, it was observed that at 5–100 mg/mL, it resulted in a 91.17–88.23% survival rate, whereas after 6 h of exposure to the treatment, it resulted in a 6.86–2.94% survival rate. As observed in Figure 3D, treatment with the extracts at 5, 10, 50, or 100 mg/mL did not decrease the viability of *A. salina* nauplii.

### 2.7. Molecular Docking Analysis

**Figure 4:**
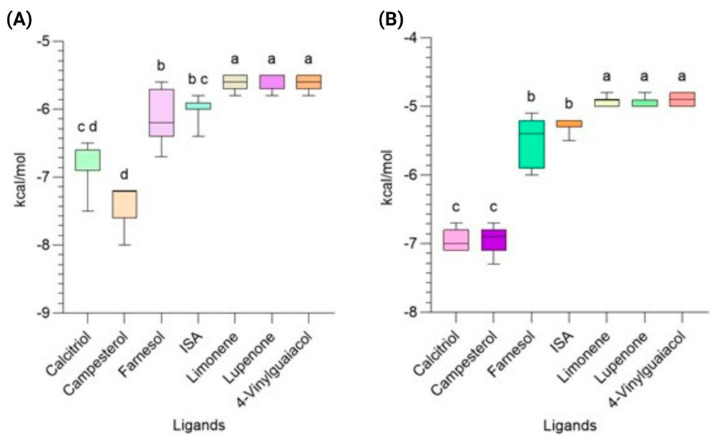
Ligand binding energies to (**A**) *E. coli* and (**B**) *S. aureus* DNA gyrases. ISA: isopropenylcyclohexanol acetate (*p* < 0.0001). Data are represented as the mean ± standard deviation (SD) from three independent experiments.

The Autodock Vina parameters were set to energy rank = 3 and exhaustiveness = 16 to obtain the optimal precision poses. The number of simulations performed for each ligand was 30 to obtain binding energy values with reduced dispersion. The compound with the most favorable binding energy against *E. coli* DNA gyrase (PDB ID: 6KZV) was campesterol at −7.44 ± 0.36 kcal/mol, followed by calcitriol at −6.82 ± 0.41 kcal/mol, suggesting that these compounds have potential activity as ligands. The binding energies of the other compounds were below −6.5 kcal/mol, indicating more moderate activity (Figure 4A). For the DNA gyrase of *S. aureus*, calcitriol and campesterol showed energies of −6.940 ± 0.16 kcal/mol and −6.940 ± 0.22 kcal/mol, respectively, indicating a similar behavior to that with *E. coli* DNA gyrase (Figure 4B).

To evaluate the differences between the ligands and the effect size (eta squared) η^2^, the assumptions of normality and homoscedasticity were validated. After this, the non-parametric Kruskal–Wallis test (H = 138.96 for DNA gyrase of *E. coli*, H = 154.767 for *S. aureus*; *p* < 0.001) and a post hoc Dunn test were performed. High H values indicate important differences between groups. The calculated η^2^ for each protein was 0.7914 and 0.8855, indicating a large effect size, which was validated by their Cohen’s f of 1.948 and 2.781 (for reference, a high value is f ≈ 0.40), suggesting practical and biological relevance. However, although the statistical analyses showed favorable results, the in vitro tests showed little to no effect; this may be due to a low effective concentration in the extracts or insufficient mechanisms of action to achieve the observable effect despite the promising in silico results.

**Figure 5:**
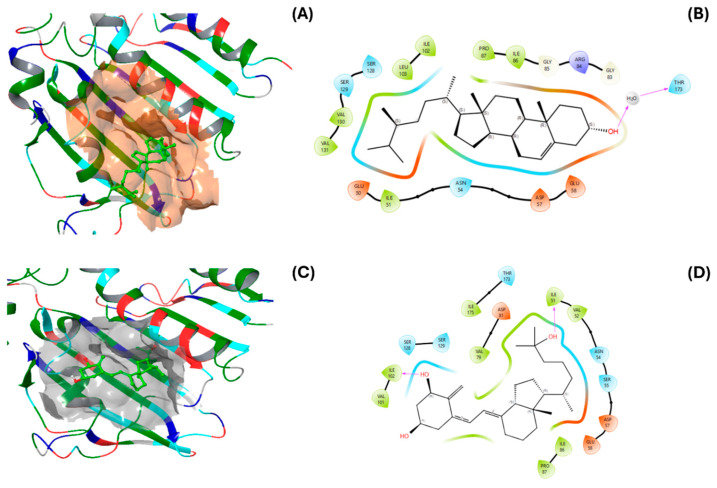
Three- and two-dimensional models of (**A**,**B**) campesterol and (**C**,**D**) calcitriol interactions with DNA gyrase from *Staphylococcus aureus*.

**Figure 6:**
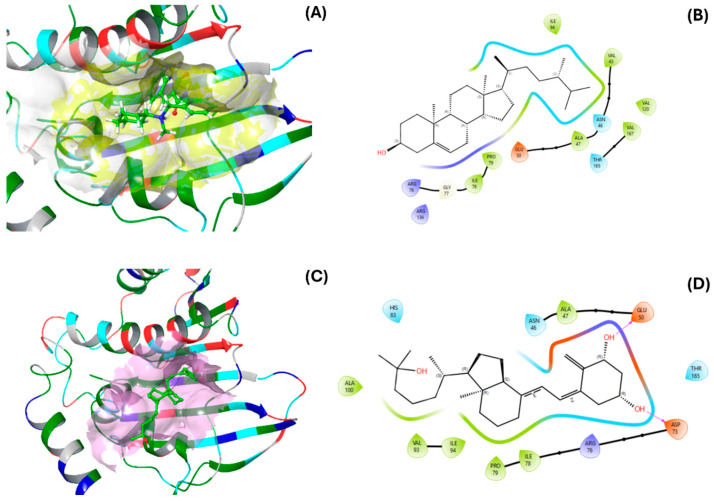
Three- and two-dimensional models of (**A**,**B**) campesterol and (**C**,**D**) calcitriol interactions with DNA gyrase from *E. coli*.

Figure 5 and Figure 6 illustrate the interactions of the ligands with the DNA gyrases of *S. aureus* and *E. coli*. For *S. aureus*, the interacting residues in its DNA gyrase include ILE102, ALA98, VAL101, ILE86, GLN91, and PRO87 (Figure 5A,B). Notably, ILE102, ILE86, and PRO87 coincide with the residues that interact with the protein and the native ligand of *S. aureus* (Figure 5C,D). Additionally, the residues in contact with calcitriol (ILE51, ASN54, GLU58, ILE86, and PRO87) overlap with ILE102, PRO87, ILE86, and GLN91 (residues that interact with the protein and the native ligand).

The interactions of campesterol with *E. coli* DNA gyrase (Figure 6A,B) were mediated by ILE94, PRO79, ILE78, GLY77, ARG76, ASN46, ALA47, GLU50, THR165, and VAL167. The interactions of the native ligand are mediated by GLU50, ARG76, PRO79, VAL120; ALA47, ILE78, ILE94, and ASP7 (Figure 6C,D), which are similar to those with ILE94, ILE78, VAL120, ALA47, and ASP73. However, the interactions with campesterol were of the Van der Waals type. Compared to calcitriol, campesterol showed a better binding profile with *E. coli* DNA gyrase, which was similar to that of the reference ligand (2-oxo-1,2-dihydroquinoline derivative, crystal 6KZV).

### 2.8. In Silico Drug-Likeness and Toxicity Predictions

An Absorption, Distribution, Metabolism, Excretion, and Toxicity (ADMET) analysis was conducted using SwissAdme and pkCSM to examine the physicochemical, pharmacokinetic, and solubility properties of the bioactive compounds (see Table 5) to assess their potential application as drugs. Molecular weight (MW), logP (consensus), solubility (computed by the ESOL model), and its corresponding classification, TPSA (polar surface area), HBAs (hydrogen bond acceptors), HBDs (hydrogen bond donors), and RotB (rotatable bonds) are the key descriptors that affect the absorption, bioavailability, and permeability of compounds. Gastrointestinal absorption (GI Absorption) predicts oral absorption potential, while sinking to violating Lipinski rule indicates how similar or dissimilar drug candidates are to the reference.

**Figure 7:**
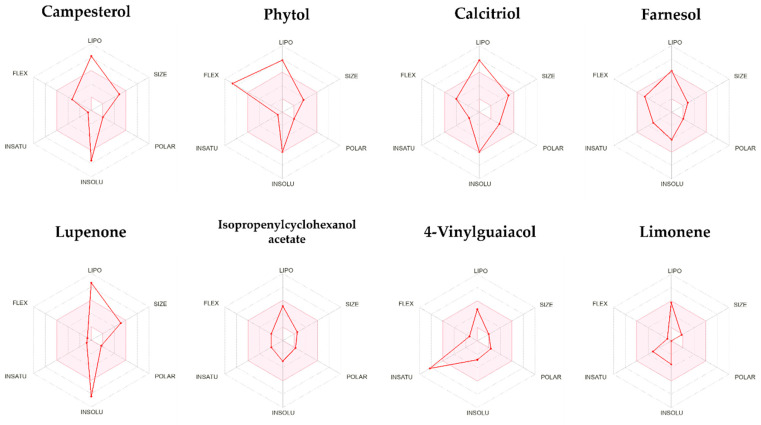
Radar plots illustrating bioavailability features based on six physicochemical properties: LIPO (lipophilicity), SIZE (size), POLAR (polarity), INSOLU (insolubility), INSATU (unsaturation), and FLEX (flexibility). The pink region indicates the physicochemical range that the radar plot of the target compound must completely occupy to be deemed drug-like.

Figure 7 presents a bioavailability radar plot that highlights six distinct physicochemical properties: lipophilicity, size, polarity, solubility, flexibility, and saturation [12].

**Figure 8:**
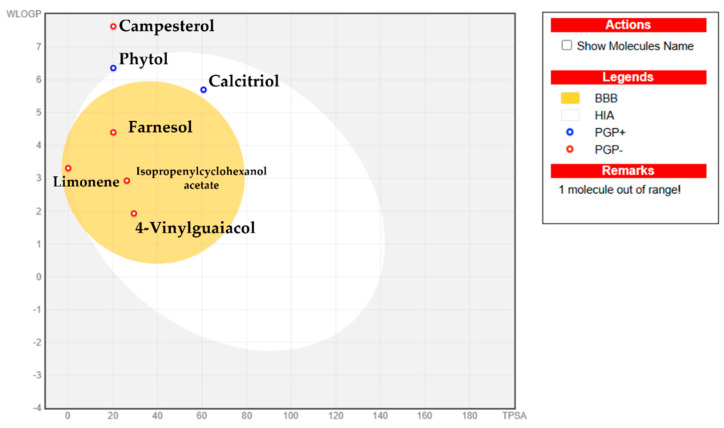
Boiled egg plot showing the water partition coefficient (WlogP) versus the topological polar surface area (TPSA) of the ligands.

Figure 8 illustrates the *in silico* bioavailability properties considering the water partition coefficient (WlogP) versus the topological polar surface area (WlogP vs. TPSA). The yellow region, the yolk, represents the zone for blood–brain barrier (BBB) permeability, while the white region corresponds to the gastrointestinal absorption (HIA) zone. Compounds located in the gray area are predicted to be unable to be absorbed through the gastrointestinal tract nor able to penetrate the BBB to reach the central nervous system (CNS).

Table 6 presents the results of the in silico toxicity analysis. As can be observed, all the analyzed pounds were found to be safe, except for calcitriol, which was classified as potentially carcinogenic and toxic, with an LD_50_ of 1 mg/kg.

## 3. Discussion

Species from the genus *Equisetum* are of great importance in TM due to their intrinsic antimicrobial, anticancer, anti-inflammatory, antidiabetic, and wound-healing properties. There is scientific evidence for the use of polar extracts of the stems or aerial parts of species from the genus *Equisetum* in TM. However, only a limited number of studies have focused on their non-polar and partially polar counterparts. In this work, the aerial parts of *E. hyemale* L. were macerated to obtain extracts, which were subsequently studied using spectroscopic and chromatographic approaches in conjunction with biological and nutritional analyses.

### 3.1. Bromatological Analysis

Bromatological analyses are used to determine the chemical composition and nutritional value of processed foods, beverages, nutraceuticals, and raw agricultural products. Currently, no studies have performed this type of analysis on *E. hyemale* L. In this study, it was used as a complementary approach for determining the nutritional value of *E. hyemale* L. extracts and validating the traditional knowledge about this species since in traditional practices, it is consumed as a tea in regions in Brazil [13]. Here, it was determined that the aerial parts of *E. hyemale* L. are contains 11.20, 18.0, 4.40, 2.50, and 38% water, ash, crude protein, ether extract, and crude fiber, respectively.

### 3.2. TPC and TFC Analyses

The TPC and TFC assays were initially used to evaluate the phytochemical composition of the ethyl acetate extract of *E. hyemale* L. Both assays were only used on the ethyl acetate extract since they can identify potential polar compounds such as phenolics and flavonoids, which are generally more soluble in polar solvents. In the case of the non-polar extracts, the TPC and TFC assays were not performed since they are more likely to contain higher concentrations of hydrophobic substances such as fatty acids. According to the retrieved regression equation (*y* = 0.0023*x* + 0.1957; *R*^2^ = 0.99), the TPC of the ethyl acetate extract of *E. hyemale* L. was 58.39 mg GAE/g, whereas the TFC of the same extract was 18.71 mg Qu/g. The TFC was assessed by considering the following regression equation: *y* = 0.0028*x* + 0.0727 (*R*^2^ = 0.993), which was obtained from a previous study [14]. The equation for determining the TPC of the ethyl acetate extract is presented in Figure S5. The calculated TPC and TFC of the ethyl acetate extract obtained in this study are challenging to compare since only one study has reported that an acetone/water-based (80:20 *v*/*v*) extract of the stems of *E. hyemale* L. contained 32.20 μg GAE/mg extract [11]. Similarly, other reports on *E. hyemale* L. have demonstrated that the TPC and TFC of a stem extract obtained with 40% ethanol were 41.9 ± 2.4 mg gallic acid equivalents per gram of sample and 5.5 ± 2.7 mg Qu/g, respectively [3]. In another study involving methanol, ethanol, and water extracts of *E. hyemale* L., it has been reported that the TPCs were 224.04 ± 2.87, 231.65 ± 2.77, and 149.84 ± 0.82. It was revealed that the TFCs of the same extracts were 9.99 ± 0.63, 12.82 ± 0.52, and 5.04 ± 2.15 mg Qu/g [15]. The variability between the reported TPC and TFC values in this study compared with other reports could be related to differences in the polarity of the solvents, the amount of raw material used for extract preparation, and the experimental conditions.

### 3.3. Chromatography Evaluation of the Extracts of E. hyemale L.

In this work, GC/MS and HPLC were employed to determine the phytochemical composition of the hexane, chloroform, and ethyl acetate extracts of *E. hyemale* L. In this study, hexane, chloroform, and ethyl acetate solvents were utilized to perform a sequential extraction to obtain a wide variety of secondary metabolites that have antibacterial, antioxidant, and cytotoxic properties. Considering their polarity, GC/MS was utilized to analyze the hexane and chloroform extracts due to their high volatile and semi-volatile compound contents. HPLC was employed for investigating the ethyl acetate extract considering its high content of heat-sensitive and polar-based compounds such as flavonoids and phenolic acids. The use of a polarity gradient during sample preparation enabled us to obtain extracts with suitable phytochemical contents to induce cancer cell death or scavenge free radicals, which could have the potential to be used in the treatment or prevention of oxidative stress-related disorders.

#### 3.3.1. GC/MS Analysis

As noted in Table 1, the hexane extract consisted of phytosterols (campesterol, ergost-5-en-(3*β*)-ol, 24-methylcholesterol, and phytol), fatty acids (9-eicosyne, 2-hexadecen-1-ol, arachidonic acid methyl ester, palmitic acid, and *n*-hexadecanoic acid), glycerolipids (1-mono-linolenin), and triterpenes (lupenone). In comparison, the chloroform extract was composed of terpenes (isopulegyl acetate, *D*-limonene, limonen-6-ol-pivalate, and retinal), phenols (*p*-vinylguaiacol and *o*-acetyl-*p*-cresol), terpenoids (5*α*-cholestan-3*β*-ol, 2-methylene, and α-retinene), fatty acids (1-heptatriacontanol), and steroids (25,26-dihydroxy-vitamin D3). Similarly to the hexane and chloroform extracts, the ethyl acetate extract contained phenols (*p*-vinylguaiacol, *o*-acetyl-*p*-cresol, and 4-hydroxy-2-methylacetophenone), terpenoids (1-dodecanol, 3,7,11-trimethyl, and *trans*-phytol), alkenes (9- and 3-eicosyne, eicosen-1-ol, cis-9, and *n*-heptacosane), fatty acids (nonadecanol, tetramethyl-2-hexadecen-1-ol, palmitic acid, and linolenic acid), sterols (*β*-sitosterol), and vitamin E (*α*-tocopherol).

The secondary metabolites identified by GC/MS in these extracts are in accordance with other reports of the phytochemical composition of polar extracts of *E. hyemale* L. For instance, it has been reported that ethanol extracts of *E. hyemale* L. contain phytosterols such as γ-sitosterol, cycloartenol, and campesterol together with phenylpropenoids including phenol, 2,4-bis (1-phenylethyl) [3]. In other studies, diethyl ether extracts of the aerial parts of *E. hyemale* L. have been reported to contain 4-vinylguaiacol and isovanillin, aromatic compounds that were also identified in the ethyl acetate extract obtained in this work [16]. The variability in the presence of other compounds in the referenced studies compared to those reported in this study can be predominantly attributed to differences in extract polarity, the type of column used, and the chromatography method. In addition to these limitations, other studies have reported different phytochemical compositions of other *Equisetum* species (e.g., *E. hyemale* L., *E. arvense*, *E. palustre*, *E. sylvaticum*, and *E. telmateia*). However, they employed HPLC–DAD-ESI-MS analyses [11]. The data from GC/MS and HPLC–DAD-ESI-MS analyses cannot be directly compared as the former focuses on volatile and semi-volatile substances. In contrast, the latter encompasses the determination of polar, thermally labile, and high-molecular-weight metabolites.

#### 3.3.2. HPLC Analysis

The polar phytoconstituents of the ethyl acetate extract were identified through an HPLC analysis. Among the compounds described previously in plants of the genera Equisetum, we detected the presence of caffeoylquinic acids and glycosidic derivatives of luteolin. 1-*O*-caffeoylquinic and 4-*O*-caffeoylquinic have been reported in a hydroalcoholic extract of *E. telmateia*, particularly sterile stems, using HPLC [1]. In the case of luteolin-7-*O*-glucoside, previous reports have found this compound in *E. arvense* L and *E. myriochaetum* using different extraction solutions, in the EtOAc fraction, and using MSPD extraction. One point to highlight is that the presence of this compound was detected using different techniques such as HPLC and CC [1].

Gallic acid and quercetin aglycone were also detected in *E. hyemale* L. in an extract prepared using a solution of ethanol in a ratio of 1:10 (powder–solvent) and using HPLC [3]. From the same plant, reverse-phase high-performance liquid chromatography detected kaempferol-3-*O*-glucoside, which was also found in our extracts, highlighting the presence of flavanols in these plant species [3]. UPLC-ESI-MS was able to detect some of the compounds detected in this study in *E. hyemale* L., such as myricetin and isorhamnetin, which may have antimicrobial effects [17]. These compounds were detected in an extract produced by first extracting the vegetable material using a hydroalcoholic extract with 70% ethanol and subsequently evaporating it to obtain an aqueous extract that was successively partitioned with dichloromethane, ethyl acetate, and *n*-butanol [17].

Some compounds like myricetin, quercetin, and kaempferol have been detected in many different *Equisetum* species, such as *E. arvense* L., *E. hyemale* L., *E. palustre* L., *E. sylvaticum* L., and *E. telmateia*, using HPLC-MS [11]. The extracts used in this study were prepared using a two-step extraction method with a mixture of acetone and water (80/20 *v/v*). The acetone from each extract was removed and the obtained aqueous extract was submitted to successive extractions with three portions of dichloromethane, ethyl acetate, and n-butanol using a separating funnel. The presence of these compounds, and particularly the absence of some gallic acid and caffeic acid components, indicate a number of shared compounds between the species in this genus but there is a strong influence from the ecological niche of each plant [11].

### 3.4. Antibacterial Activity Analysis

Antibacterial resistance poses a significant challenge to healthcare systems. The World Health Organization (WHO) classifies multidrug-resistant bacterial strains into critical and high-priority categories. Here, the antibacterial activity of extracts of *E. hyemale* L. was analyzed against a panel of critical and high-priority bacteria strains: *Staphylococcus aureus*, *Escherichia coli*, *Klebsiella pneumoniae*, *Pseudomonas aeruginosa*, and *Proteus mirabilis*. As noted in Table S2, treatment with the extracts did not inhibit the growth of the cultured strains at the tested concentrations. Despite their phytochemical content, the poor activity of the hexane, chloroform, and ethyl acetate extracts of *E. hyemale* L. could be related to compound bioavailability and concentration factors, as well as possible antibacterial mechanisms [18]. For example, it has been reported that the molecular size, weight, polarity, and solubility of certain secondary metabolites can prevent them from entering into bacterial cells, resulting in diminished antibacterial activity. It has also been documented that the genetic adaptation and efflux capability of drug-resistant bacteria strains can decrease the intracellular concentration of antibacterial compounds, conferring protection and resistance [19].

Despite the weak antibacterial activity of the reported extracts in this work, recent studies have demonstrated that ethanol extracts of the stems of *E. hyemale* L. can inhibit the growth of *S. aureus* and *E. coli*, with minimum inhibitory concentrations (MICs) of 14.40 and 27.60 mg/mL after 48 h of exposure, respectively [3]. On the other hand, it has been reported that hydroalcoholic extracts and fractions (dichloromethane, ethyl acetate, and *n*-butanol) of the stems of *E. hyemale* L. can inhibit the growth of *P. aeruginosa* (with an MIC of 26.20–13.10 μg/mL), reduce biofilm formation by 29–64%, and produce violacein [20]. Here, it was noted that treatment with 5, 10, 50, and 100 μg/mL did not inhibit the growth of the tested strains. The differences between the results of this study and other reports can be attributed to variations in experimental design, the use of logarithmic scales to present results, the tested concentrations, and the raw materials used for extract preparation.

### 3.5. Cytotoxicity Analysis

In this work, the cytotoxicity of the hexane, chloroform, and ethyl acetate extracts of *E. hyemale* L. was investigated using representative cell line models of colon cancer (CACO-2), hepatocellular cancer (HEPG-2), fibroblasts (HDF-1), and macrophages (RAW 264.7). Cytotoxicity evaluations of extracts of *E. hyemale* L. are limited since only a few studies tested cancer or healthy cell lines. For instance, it has been reported that extracts of the overground parts of *E. hyemale* L. obtained using ethanol via microwave-assisted extraction can decrease the proliferation of L1210 cells, a lymphocytic leukemia cell line, in a dose- and time-dependent manner by arresting their cell cycle at the G2/M phase and causing chromatin condensation, DNA damage, downregulation of the mitochondrial membrane potential, and phosphatidylserine exposure [5]. Similarly, it has been documented that an ethanol crude extract of the stems of *E. hyemale* L. and its *n*-hexane, dichloromethane, and ethyl acetate fractions can cause the death of oral tumor cell lines (i.e., SCC-4, SCC-9, and SCC-25) through apoptosis, chromatin condensation, activation of caspases 3/7, and DNA fragmentation. In the same study, it was found that treatment with ethyl acetate fractions can reduce the volume and weight of tumor-bearing SCC-9 xenotransplants while causing lymphocytic infiltration and arterial and venous hyperemia at concentrations of 300 and 600 mg/kg [6].

According to the National Cancer Institute, the cytotoxicity of the hexane and ethyl acetate extracts against the CACO-2 cell line was weak, as indicated by their LC_50_ values of 189.55 and 284.27 μg/mL. Conversely, the chloroform extract can be considered non-cytotoxic (595.78 μg/mL). Similarly, it was found that the cytotoxicity of the hexane, chloroform, and ethyl acetate extracts against the HEPG-2 and HDF-1 cell lines was moderate. The calculated LC_50_ values were 283.37, 176.08, and 252.21 μg/mL, respectively. For the HDF-1 cell line, the LC_50_ values were 139.60, 89.49, and 256.89 μg/mL. Similarly, the cytotoxicity of the extracts in this study was moderate against the RAW 264.7 cell line, with LC_50_ values of 196.42, 152.22, and 276.22 μg/mL.

Although other research approaches are required to confirm the cytotoxic mechanisms of the hexane, chloroform, and ethyl acetate extracts of *E. hyemale* L., their activity towards the CACO-2 and HEPG-2 cell lines can be attributed to their phytochemical content. In the case of the hexane extract, it has been reported that lupenone and its synthetic derivatives can induce the death of prostate cancer cells (e.g., PC3 and DU145) by upregulating the activation of caspases 3, 8, and 9, thereby promoting apoptosis and autophagy-mediated cell death [21]. Moreover, the cytotoxicity of the chloroform extract might have resulted from the activity of *D*-limonene, which has been demonstrated to induce cancer cell death by increasing the expression of pro-apoptotic proteins (e.g., Bax), inhibiting the Ras/Raf/ERK1/2 and PI3K/Akt signaling pathways, arresting the cell cycle, and enhancing the levels of cytochrome C [22]. The activity of the ethyl acetate extract against the cultured cell lines in this study may be related to vinylguaiacol, which has been reported to induce apoptosis and chromatin condensation in colon cancer cell lines, including HCT-116 and HT-29 [23]. In this study, treatment with the hexane, chloroform, and ethyl acetate extracts also decreased the viability of the HDF-1 and RAW 264.7 cell lines, which has also been observed with extracts of *Melianthus comosus* Vahl, *Tetradenia riparia*, and *Warburgia salutaris* [24,25].

### 3.6. Antioxidant Analysis

Here, it was noted that treatment with the hexane, chloroform, and ethyl acetate extracts of *E. hyemale* L. inhibited the formation of DPPH radicals, with IC_50_ values of 303.53, 913.99, and 1111.10 μg/mL, respectively. Against H_2_O_2_ radicals, the extracts were able to scavenge them, with IC_50_ values of 118.44, 6.40, and 121.71 μg/mL, respectively. Among the tested radicals, the extracts exhibited a strong capability to scavenge ABTS radicals, with IC_50_ values of 28.68, 2.64, and 2.57 μg/mL, respectively. The inhibition of free radical formation by the extracts can be attributed to their campesterol, ergost-5-en-3β-ol, *p*-vinylguaiacol, 25,26-dihydroxyvitamin D3, sitosterol, or tocopherol content, which, due to the presence of hydroxyl groups, can donate electrons to neutralize the formation of the tested free radicals. These findings are challenging to compare with other studies as this is the first time that three antioxidant assays were conducted to analyze the scavenging capacity of hexane-, chloroform-, and ethyl acetate-based preparations of *E. hyemale* L. Only one study reported that ethanol and methanol extracts of *E. hyemale* L. can scavenged DPPH radicals, with IC_50_ values of 22.53 and 27.36 μg/mL, respectively [15]. The difference between the IC_50_ values from this study and the results provided in Table 4 could be due to the polarity of the extracts. In the case of the IC_50_ values calculated against ABTS, it should be noted that ABTS possesses higher reactivity towards potential hydrophilic and lipophilic antioxidants due to their hydroxyl or carboxyl functional groups, which can scavenge free radicals through hydrogen atom donation or electron withdrawal phenomena [26].

### 3.7. In Vivo Toxicity Analysis

Here, it was reported that treatment with the hexane, chloroform, and ethyl acetate extracts of *E. hyemale* L. did not decrease the viability of *A. salina* nauplii, suggesting that they are biocompatible at the tested concentrations (5, 10, 50, and 100 μg/mL). Although this is the first time the toxicity of extracts of *E. hyemale* L. has been evaluated in vivo, the results are consistent with extracts of *E. arvense*, which have been demonstrated to be non-toxic, with an LC_50_ greater than 1 mg/mL against *A. salina* [27].

*C. elegans* nematodes are transparent, cylindrical organisms with a well-defined body; they are frequently used for developmental biology, neurobiology, and aging research applications. Here, it was determined that treatment with the extracts of *E. hyemale* L. can decrease the viability of *C. elegans* nematodes in a dose- and time-dependent manner. Similar to *A. salina*, this is the first time *C. elegans* was utilized to analyze the toxicity of extracts of *E. hyemale* L.; however, it aligns with our research program, which involves using *C. elegans* nematodes to evaluate oxidative stress resistance and the antioxidant activity of tea-based beverages [28] and powders from raw natural materials (*Opuntia ficus indica*) [29]. It was noted that the extracts decreased the viability of *C. elegans* nematodes after 6 h of exposure to the treatment, which can be attributed to the capability of the secondary metabolites to enhance ROS generation, disrupt metabolic processes, and induce severe detrimental changes at cellular or physiological level [30]. In comparison with *A. salina*, *C. elegans* possesses a more complex nervous and physiological system, making it susceptible to toxins that affect cellular processes [31]. As listed in Table S3, the LC_50_ values of the hexane, chloroform, and ethyl acetate extracts of *E. hyemale* L. towards *C. elegans* varied in a time- and concentration-dependent manner, where the predominant toxicity was observed after 3–6 h of exposure to the treatment. Further studies are needed to determine the effect of treatment with these extracts on the molecular and biochemical features of *C. elegans*.

### 3.8. Molecular Docking Analysis and In Silico Drug-Likeness and Toxicity Predictions

The in silico analysis presented in this study was based on the main compounds identified in the hexane, chloroform, and ethyl acetate extracts of *E. hyemale* L. The phytoconstituents (campesterol and calcitriol) of the extracts of *E. hyemale* L. showed high affinity towards the DNA gyrases of *S. aureus* and *E. coli*. These results can be attributed to the fact that in silico models assess the individual interaction between secondary metabolites and bacterial targets utilizing computation. Thus, the insights provided from in silico modeling do not account for the experimental complexity of in vitro assays and only yield significant information about the potential mechanisms of action of individual compounds to identify promising candidates for the development of antibacterial drugs.

As noted in Table 6, the results suggested variations in the compounds’ lipophilicity, solubility, and intestinal permeability, which are essential parameters to determine their pharmacological potential. Regarding lipophilicity, compounds such as campesterol, calcitriol, lupenone, farnesol, and phytol showed high logP values (consensus > 6), indicating a high affinity with lipid compounds. This high affinity can increase passive permeability across membranes while also decreasing solubility in water. On the other hand, some compounds, such as 4-vinylguaiacol, isopropenylcyclohexanol acetate, and limonene, exhibited better solubility profiles, possibly due to their lower molecular weights and higher polarities. Regarding their pharmacokinetic properties, most compounds showed relatively low absorption through the gastrointestinal (GI) tract, except for 4-vinylguaiacol and isopropenylcyclohexanol acetate, which were predicted to have a high probability of absorption. This is due to a relatively smaller number of heavy atoms and a lower topological polar surface area (TPSA). None of the compounds were identified as inhibitors of CYP enzymes, which would significantly minimize the likelihood of drug interaction.

Most compounds are poorly absorbed in the GI tract [32], except for 4-vinylguaiacol, limonene, and isopropenylcyclohexanol acetate, which are highly absorbable. This may be due to the lower number of atoms, higher molecular weight, and low TPSA. Their inability to inhibit the CYP3A4 enzyme is desirable in terms of pharmacokinetic safety, since it plays a role in the metabolism of 75 to 90% of commercial drugs [33], which translates into a low potential for interference with other drugs (see Table 6). According to Figure 7, it was also observed that farnesol, isopropenylcyclohexanol acetate, and limonene are found within the pink area, indicating acceptable oral bioavailability. Campesterol, calcitriol, phytol, and lupenone exhibit reduced water solubility, which negatively impacts their oral bioavailability. Advanced formulations are required to improve this characteristic.

As shown in Figure 8, farnesol, isopropenylcyclohexanol acetate, 4-Vinylguaiacol, and limonene have the ability to cross the BBB. In contrast, phytol and calcitriol are able to cross the HIA zone. On the other hand, campesterol was positioned outside the egg region, indicating its inability to penetrate the BBB due to a high LogP value. Additionally, phytol and calcitriol exhibited active efflux from the CNS and the gastrointestinal lumen, mediated by P-glycoprotein (PGP+), as indicated by the blue dot. In contrast, the remaining compounds were classified as non-substrates of P-glycoprotein (PGP−), represented by the red dot. Both gastrointestinal absorption (GIA) and CNS penetration are critical factors for biomolecules to advance to pharmaceutical or clinical trials. Specifically, BBB penetration is essential for evaluating CNS effects as compounds targeting the CNS must cross the BBB, while those that are not intended to target the CNS should avoid interaction to prevent adverse effects [34,35].

## 4. Materials and Methods

### 4.1. Plant Collection and Identification

*E. hyemale* L. was collected in Multiviveros Cabrera, Atlixco, Puebla (18°55′15″ N, 98°26′41″ W). Dr. Amparo Bélgica Cerón-Carpio identified the specimens, and they were deposited in the Jardín Botánico Universitario of the Benemérita Universidad Autónoma de Puebla (BUAP; 24 Sur Av. San Claudio, Col. San Manuel C.P. 72570 Puebla de Zaragoza, Puebla, México) under voucher number 88262.

### 4.2. Bromatological Analysis

The humidity, ash, and protein contents of the *E. hyemale* L. raw material were determined following the Association of Official Analytical Chemists (AOAC) methods. Briefly, humidity was calculated after drying in an oven at 105 °C for 24 h (TERLAB^®^ model T-H-45DM, Feligneo, Zapotlán, Mexico), whereas the ash content was determined utilizing a muffle furnace at 550 °C for 4 h (TERLAB^®^ model T-M12D; Feligneo, Zapotlán, Mexico) and ether extract was analyzed with petroleum ether in a Soxhlet apparatus (ECOSHEL® modelo EXTRACTION-06C, Ecoshel Technology Ltd., Pharr, TX, USA). Crude protein was calculated based on total nitrogen determination using the Microkjeldahl technique using a DGM K-630 digester and a DMK-650 FIGURSA^®^ distiller (Figursa Industries, Cuautitlán Izcalli, Mexico). Crude fiber was analyzed by acid (H_2_SO_4_ 0.255 N) and alkaline (NaOH 0.313 N) hydrolysis.

### 4.3. Extract Preparation

The aerial parts of *E. hyemale* L. were utilized for extract preparation. Initially, they were air-dried at room temperature for three weeks. Then, 930 g was powdered using a mechanical blender and progressively macerated with hexane, chloroform, and ethyl acetate for 72 h at room temperature. The solvents were evaporated under reduced pressure to dryness using a Heidolph Laborota 4000 rotary evaporator (Schwabach, Germany). After the solvents were completely removed, the extracts were collected and stored under refrigeration until further analysis.

### 4.4. TPC and TFC Analyses

The TPC and TFC of the ethyl acetate extract of *E. hyemale* L. were determined as reported [36,37]. For the TPC assay, 300 μL of the Folin–Ciocalteu reagent (1:1 *v*/*v*) was mixed with 7.5 mL of distilled water and 1 mL of diluted ethyl acetate extract (1:100). The mixture was incubated for 3 min. Then 1 mL of sodium carbonate (20% *w*/*v*) was added. After 20 min, the absorbance of the sample was determined at 760 nm utilizing a Cary 60 UV–vis spectrophotometer (Agilent Technologies, Santa Clara, CA, USA). The TPC of the ethyl acetate was expressed in milligrams of gallic acid (GAE) equivalents per gram of sample (mg GAE/g). For the TFC assay, 100 μL of ethyl acetate extract (1 mg/mL) was mixed with 100 μL of 2% aluminum chloride (AlCl_3_). The sample was incubated for 10 min, and its absorbance was measured using the same spectrophotometer at 420 nm. The TFC of the ethyl acetate extract was assessed as a percentage of total quercetin (Qu) equivalents per gram of extract (mg Qu/g). All experiments were performed in triplicate.

### 4.5. Chromatography Evaluation of the Hexane, Chloroform, and Ethyl Acetate Extracts

#### 4.5.1. GC/MS Analysis

An Agilent Technologies 6850N gas chromatograph (Agilent Technologies, Palo Alto, CA, USA) equipped with an Agilent 5975 C mass spectrometer detector (Agilent Technologies) was employed to determine the chemical composition of the hexane and chloroform extracts of *E. hyemale* L. Briefly, samples were injected into an HP-5MS column (5% phenyl methyl siloxane, 30.0 m × 250 µm × 0.25 µm nominal; Agilent Technologies). Helium served as the carrier gas, which was maintained at a flow rate of 1 mL/min. The injection volume was 1 mL, the injector temperature was 250 °C, and the injector operated in the 10:1 split mode. Following a published method [38], the analysis was performed by setting the column oven at 60 °C for 2 min, which was ramped up to 250 °C. Individual components of the extracts were identified by comparing their retention times and fragmentation patterns with those in the National Institute of Standards and Technology Mass Spectral (NIST-MS) database. The total area of the peaks was assessed to determine their relative percentages.

#### 4.5.2. HPLC Analysis

The HPLC analysis of the ethyl acetate extract of *E. hyemale* L. was determined using published protocols [36]. Initially, 10 mL of the extract, previously dissolved in a mixture of water and acetonitrile, was injected into an Agilent Technologies 1200 series instrument equipped with a diode array detector. After this, it was analyzed utilizing a RP-18 Zorbax column (150 mm × 4.6 mm, 3.5 μm) (Agilent Technologies, Santa Clara, CA, USA) under a mobile phase consisting of water acidified with 0.1% formic acid (A) and acetonitrile (B). The absorbance was investigated at 254 and 365 nm. The polarity gradient was 0–20 min (0–20% B), 20–40 min (20–22% B), 40–43 min (22–30% B), 43–45 min (30–100%B), and 45–50 min (100% B). The secondary metabolites were identified by comparing their retention times with available literature on the phytochemistry of extracts of *E. hyemale* L.

### 4.6. Strains, Culture Media, and Antibacterial Assay

The antibacterial activity of the hexane, chloroform, and ethyl acetate extracts of *E. hyemale* L. was analyzed against a panel of pathogenic Gram-positive and Gram-negative bacteria. The Gram-positive bacteria was *Staphylococcus aureus* (ATCC 23235). The Gram-negative bacteria were *Escherichia coli* (ATCC 25922), *Pseudomonas aeruginosa* (ATCC 27853), *Klebsiella pneumoniae* (ATCC 10031), and *Proteus mirabilis* (ATCC 25933). The bacteria were cultured in Mueller–Hinton (MH) broth at 37 °C under orbital shaking using a thermostatic water bath (Julabo GmbH, Seelbach, Germany) and adjusted to 0.5 according to the McFarland scale using fresh and sterile MH broth. The Kirby–Bauer method was employed to assess the effects of the hexane, chloroform, and ethyl acetate extracts of *E. hyemale* L. on the Gram-positive and Gram-negative bacterial strains. Briefly, 100 μL of a Gram-positive or Gram-negative strain was inoculated onto Petri dishes containing MH agar. Then, 6 mm sterile disks were placed on top and impregnated with 20 µL of the hexane, chloroform, or ethyl acetate extract of *E. hyemale* L. at concentrations of 5, 10, 50, and 100 µg/mL. The Petri dishes were incubated at 37 °C for 24 h. Sterile disks impregnated with fosfomycin were appraised as a positive control, whereas sterile disks without treatment were used as a negative control. The zone of inhibition (ZOI) was evaluated utilizing a Vernier caliper. All experiments were executed in triplicate.

### 4.7. Cell Culture and Cytotoxicity Assay

The CACO-2 (ATCC HTB-37), RAW 264.7 (ATCC TIB-71), HEPG-2 (ATCC HB-8065), and HDF-1 (PCS-201-012) cell lines were cultured in Dulbecco’s Modified Eagle Medium (DMEM) F-12 containing 10% fetal bovine serum and maintained under a humidified atmosphere at 37 °C under 5% CO_2_. The cytotoxicity of the hexane, chloroform, and ethyl acetate extracts of *E. hyemale* L. was tested using the (3-[4,5-dimethylthiazol-2-yl]-2,5 diphenyl tetrazolium bromide) (MTT) assay based on published reports from our research group. Briefly, 15,000 cells were dispensed into a 96-well plate together with 100 μL of DMEM and incubated for 24 h in a humidified atmosphere at 37 °C supplemented with 5% CO_2_. The next day, the medium was removed, the wells were washed with 100 μL of phosphate-buffered saline solution, and fresh medium was added together with 5, 10, 50, and 100 μg/mL of the *E. hyemale* L. extracts. The plate was incubated overnight under the same conditions. After this, 60 μL of a working solution of MTT (5 mg/mL) was added, the formazan crystals were dissolved with dimethyl sulfoxide, and the absorbance was determined at 570 nm utilizing a Synergy HTX Multi-Mode Microplate Reader (BioTek Instruments, Winooski, VT, USA). The lethal concentration 50 (LC_50_) was calculated based on the percentage of dead cells and the utilized concentrations of each extract. The passage number for the cells used in all cytotoxicity assays was 8–10. All experiments were performed in triplicate.

### 4.8. Antioxidant Activity

The antioxidant activity of the hexane, chloroform, and ethyl acetate extracts of *E. hyemale* L. was studied using the DPPH, ABTS, and H_2_O_2_ assays. For the DPPH assay, 4 mg of DPPH reagent was dissolved in 100 mL of technical-grade ethanol and kept under constant agitation for 2 h. Then, 40 μL of the hexane, chloroform, or ethyl acetate extract at concentrations of 5, 10, 50, or 100 µg/mL was mixed with 200 μL of DPPH solution. The samples were maintained in a dark place for 30 min and analyzed in 1 cm quartz cuvettes at 517 nm using a Cary 60 UV–Vis spectrophotometer (Agilent Technologies, Santa Clara, CA, USA). For the ABTS assay, 6 mg of ABTS reagent was mixed with potassium persulfate in 100 mL of distilled water and kept under constant agitation for 30 min. Then, 40 μL of the hexane, chloroform, or ethyl acetate extract at concentrations of 5, 10, 50, or 100 µg/mL was mixed with 400 μL of ABTS solution, incubated in a dark place for 6 min, and evaluated in 1 cm quartz cuvettes at 734 nm utilizing the same spectrophotometer. For the H_2_O_2_ assays, 70 μL of H_2_O_2_ solution (40 mmol/L) was mixed with 100 μL of the hexane, chloroform, or ethyl acetate extract at concentrations of 5, 10, 50, or 100 µg/mL, and maintained in a dark place for 30 min. The absorbance of the samples was measured at 230 nm using the same spectrophotometer. Quercetin (Qu) was used as a positive control for the DPPH, ABTS, and H_2_O_2_ assays, and the half-maximal inhibitory concentration (IC_50_) was calculated based on the percentage of inhibited free radicals. All experiments were carried out in triplicate.

### 4.9. In Vivo Toxicity

The toxicity of the hexane, chloroform, and ethyl acetate extracts of *E. hyemale* L. towards *A. salina* nauplii and *C. elegans* nematodes was evaluated. For the toxicity assay against *A. salina*, dried cysts were cultured in 1 L of distilled water containing 35 g of artificial sea salt. The hatching of the cysts was promoted by adjusting the temperature to 30 °C and implementing moderate agitation. After 48 h, 10 nauplii were placed into a 96-well plate together with 250 μL of distilled water. Then, they were treated with 5, 10, 50, or 100 µg/mL of the hexane, chloroform, and ethyl acetate extract. The effect of treatment was monitored over 24 h using a Leica DMi1 inverted microscope (Wetzlar, Germany) integrated with a FLEXACAM C1 camera. For the toxicity assay using *C. elegans*, nematodes (kindly provided by the Department of Chemical and Biological Sciences from the Universidad de las Américas Puebla; strain N2, Bristol wild type) were cultured at 22 °C on Petri dishes (100 mm × 15 mm) and fed with 600 µL of the *E. coli* OP50 strain. The synchronization of nematodes was performed by washing them with M9 buffer, transferring them to 2 mL Eppendorf tubes, and centrifuging them at 4600 rpm and 4 °C for 1 min. After removing the supernatant, 1 mL of fresh M9 buffer was added, and centrifugation was implemented again. The supernatant was discarded, and 1 mL of 1 M NaOH was added to the tubes, which were then vortexed for 30 s (Vortex-Gene 2 G560, Scientific Industries, Bohemia, NY, USA). The supernatant was discarded, and pellet was resuspended with 500 µL of 1 M NaOH and a mixture of NaOH and 5% NaClO. The samples were vortexed for 60 s and centrifuged. The supernatant was removed, and the pellet was washed twice with 1 mL of M9 buffer and then centrifuged at 5600 rpm and 4 °C for 1 min each time. The resulting pellet, containing eggs, was resuspended and placed onto Petri dishes containing *E. coli* OP50. The Petri dishes were incubated for 3 d. Similar to the toxicity assay against *A. salina*, 100 µL of M9 buffer was added to a 96-well plate together with 20 µL of L4 nematodes. Then, the hexane, chloroform, and ethyl acetate extracts were added at a concentration of 5, 10, 50, or 100 µg/mL. The plate was incubated at 22 °C, and the effect of the treatment with the extracts was observed utilizing an inverted microscope.

### 4.10. Molecular Docking Analysis

Autodock Vina was used for the molecular docking studies. The exhaustiveness parameter was set to 16 since higher values offer significantly better performance, which is particularly useful for molecular dynamics studies. The energy rank was set to 3 since this is the default value for the energy difference between poses. The structure data files (sdf) from PubChem were downloaded and optimized in Avogadro using the Molecular Mechanics Force Field 94 (MMFF94) and Steepest Descent algorithms that consider bond stretching, bond angles, bond twists, Van der Waals interactions, and partial charges to minimize the energy of the compounds before orienting them to the sites of interest on the protein [39]. The crystal structures of the DNA gyrases of *E. coli* (PDB ID: 6KZV) and *S. aureus* (PDB ID: 4P8O) were obtained from the RCSB protein repository. In Autodock 4, they were processed by removing water molecules, repairing missing bonds, and adding the charges of Kollman. The ligand grid box was defined around the native ligands of each crystal: aminobenzimidazole urea [40], and a 2-oxo-1,2-dihydroquinoline derivative [41].

### 4.11. In Silico Drug-Likeness and Toxicity Predictions

The SwissADME web tool and pkCSM were utilized to assess the ADMET profile. The ProTox-III server was used to evaluate the toxicological risks linked to specific compounds. This platform integrates molecular similarity, pharmacophores, fragment propensities, and machine learning models to predict various toxicity endpoints.

### 4.12. Statistical Analysis

Data retrieved from the experiments were analyzed using one-way ANOVA followed by Tukey’s mean separation test. The OriginPro 2023 software (OriginLab, Northampton, MA, USA) was employed for data processing.

## 5. Conclusions

This study demonstrated the extraction of the aerial parts of *E. hyemale* L. using maceration and hexane, chloroform, and ethyl acetate solvents. The GC/MS and HPLC evaluations revealed that these extracts are important sources of fatty acids and glycosidic derivatives of flavonoids with significant implications for the biomedical pipeline. Even though the findings of this work demonstrated that these extracts exhibit limited anticancer activity, treatment with these extracts did decrease the viability of CACO-2 (LC_50_ 189.55–595.78 μg/mL) and HEPG-2 (LC_50_ 176.06–283.57 μg/mL) cells, as well as HDF-1 (LC_50_ 89.49–256.89 μg/mL) and RAW 264.7 (LC_50_ 152.22–276.22 μg/mL) cells. This work also showed that the hexane, chloroform, and ethyl acetate extracts of *E. hyemale* L. have a strong scavenging capacity for ABTS radicals (LC_50_ 2.57–2.68 μg/mL), moderate activity against H_2_O_2_ radicals (LC_50_ 89.49–256.89 μg/mL), and a poor antioxidant capability towards DPPH radicals (LC_50_ 303.53–1111.10 μg/mL). The toxicity results revealed that treatment with these extracts is not toxic to *A. salina* nauplii, but highly toxic to *C. elegans* nematodes. Molecular docking evaluations demonstrated high binding energies between secondary metabolites from *E. hyemale* L. and the DNA gyrases of *E. coli* and *S. aureus*, suggesting possible mechanisms of action. ADMET profiling revealed that compounds such as isopropenylcyclohexanol acetate have high gastrointestinal absorption, whereas compounds such as farnesol, limonene, and 4-vinylguaiacol can pass through the BBB. In contrast to the other evaluated compounds, it was noted that calcitriol can be considered highly carcinogenic as it was classified as a type 1 toxicity agent. The results of this work validate the biological effects of non-polar and polar extracts of the aerial parts of *E. hyemale* L. and expands the knowledge on their pharmacokinetic and toxicological potential. It should be noted that, even though the hexane and chloroform extracts displayed limited anticancer activity, they contained a wide array of secondary metabolites that can be leveraged for the development of anticancer formulations. Still, other approaches should be used to diminish their toxicity towards in vitro and in vivo models such as HDF-1 and *C. elegans*. The hexane and chloroform extracts represent potential antibacterial formulations due to their high campesterol and calcitriol contents, which may interact with the main structural components of challenging bacteria such as *S. aureus* and *E. coli*. Given this, future studied should test extracts of *E. hyemale* L. on models with higher molecular, cellular, and biological complexity or integrate the extracts with novel approaches to improve their efficacy while reducing their toxicity.

## Figures and Tables

**Table 1:** GC/MS analysis of the hexane, chloroform, and ethyl acetate extracts of *E. hyemale* L.

Extract	Rt (min)	R Match	Match	Compound
Hexane	6.966	877	825	Campesterol
	6.971	858	809	Ergost-5-en-(3*β*)-ol
	8.162	882	852	24-Methylcholesterol
	10.439	810	800	2-Oleyloxy-1-ethanol
	10.462	819	808	3-Eicosyne
	10.742	950	821	2-Hexadecen-1-ol
	10.954	946	841	Unknown
	10.977	945	877	Phytol
	10.982	829	801	9-Eicosyne
	11.257	858	827	Arachidonic acid methyl ester
	11.555	883	819	Palmitic acid
	12.012	816	758	1-Mono-linolenin
	12.058	822	785	Lupenone
	12.276	917	898	*n*-Hexadecanoic acid
Chloroform	6.531	911	767	Isopulegyl acetate
	6.525	799	788	*D*-limonene
	7.692	886	807	*p*-Vinylguaiacol
	7.698	905	822	*o*-Acetyl-*p*-cresol
	8.230	783	762	Limonen-6-ol-pivalate
	8.304	846	737	Retinal
	8.419	831	808	5*α*-Cholestan-3*β*-ol, 2-methylene
	9.392	788	779	1-Heptatriacontanol
	9.46	852	732	α-Retinene
	9.512	790	750	25,26-Dihydroxy-vitamin D3
Ethyl acetate	23.554	926	919	*p*-Vinylguaiacol
	23.806	884	868	*o*-Acetyl-*p*-cresol
	24.149	841	813	4-Hydroxy-2-methylacetophenone
	35.839	847	809	1-Dodecanol, 3,7,11-trimethyl
	35.930	830	828	*trans*-Phytol
	35.953	823	817	9-Eicosyne
	35.993	823	813	3-Eicosyne
	36.073	830	810	Nonadecanol
	36.457	825	795	Eicosen-1-ol, cis-9
	36.880	946	851	Tetramethyl-2-hexadecen-1-ol
	38.751	858	814	Palmitic acid
	42.133	830	796	Linolenic acid
	43.764	849	797	*β*-Sitosterol
	47.323	910	868	*n*-Heptacosane
	49.606	823	785	*α*-Tocopherol

Abbreviations: Rt, retention time; R match, match factor.

**Table 2:** HPLC analysis of the ethyl acetate extract of *E. hyemale* L.

Extract	Rt (min)	Compound	Reference
Ethyl acetate	1.212	1-*O*-Caffeoylquinic acid	[1]
	1.329	4-*O*-Caffeoylquinic acid	[1]
	1.424	Gallic acid	[3]
	8.880	Caffeic acid 4-*O*-glucoside	[3]
	9.255	Luteolin-7-*O*-glucoside	[1]
	10.116	Quercetin-3-*O*-rutinoside	[1]
	10.978	Quercetin 3-*O*-glucoside	[1]
	11.349	Apigenin 5-*O*-glucoside	[1]
	11.806	Kaempferol-3-*O*-glucoside	[3]
	12.499	Quercetin aglycone	[3]
	12.577	Myricetin	[10]
	13.203	Kaempferol aglycone	[11]
	13.582	*p*-Hydroxybenzoic acid *O*-hexoside	[11]
	14.232	3′,4′-Dihydroxypropiophenone-3-*O*-glucoside	[11]
	14.440	3′-*O*-methylquercetin	[3]
	14.950	Luteolin aglycone	[11]
	15.195	Apigenin	[3]
	15.929	Caffeoylputrescine	[11]
	16.612	Isorhamnetin	[10]

Abbreviations: Rt, retention time.

**Table 3:** LC_50_ of the hexane, chloroform, and ethyl acetate extracts of *E. hyemale* L. against the CACO-2, HEPG-2, HDF-1, and RAW 264.7 cell lines. Concentrations are expressed in μg/mL.

Extract	CACO-2	HEPG-2	HDF-1	RAW 264.7
Hexane	189.55	283.37	139.60	196.42
Chloroform	595.78	176.08	89.49	152.22
Ethyl acetate	284.27	252.21	256.89	276.22

**Table 4:** IC_50_ of the hexane, chloroform, ethyl acetate extracts of *E. hyemale* L. and quercetin (Qu) against DPPH, ABTS, and H_2_O_2_ radicals. Concentrations are expressed in μg/mL.

Extract	DPPH	ABTS	H_2_O_2_
Hexane	303.53	2.68	118.44
Chloroform	913.99	2.64	6.40
Ethyl acetate	1111.10	2.57	121.71
Qu	70.57	1.75	97.52

**Table 5:** ADMET analysis of compounds in the extracts of *E. hyemale* L.

Molecule	MW	logP	ESOL	TPSA	HBAs	HBDs	RotB	CYP3A4 Inhibitor	GI Absorption	Viol. Lipinski
Campesterol	400.68	6.92	Poorly soluble	20.23	1	1	5	No	Low	1
Phytol	296.53	6.25	Moderately soluble	20.23	1	1	13	No	Low	1
Calcitriol	416.64	6.25	Moderately soluble	60.69	3	3	6	No	Low	1
Farnesol	222.37	6.25	Moderately soluble	20.23	1	1	7	No	Low	1
Lupenone	426.72	7.37	Poorly soluble	17.07	1	0	1	No	Low	1
Isopropenylcyclohexanol acetate	196.29	2.85	Soluble	26.30	2	0	3	No	High	0
4-Vinylguaiacol	150.17	2.14	Soluble	29.46	2	1	2	No	High	0
Limonene	136.23	3.37	Soluble	0.00	0	0	1	No	Low	0

**Table 6:** Toxicity predictions of compounds in the extracts of *E. hyemale* L.

Ligand	LD_50_ (mg/kg)	Toxicity Class *	Cytotoxicity	Mutagenicity	Carcinogenicity	Hepatotoxicity
Campesterol	890	4	Inactive	Inactive	Inactive	Inactive
Phytol	5000	5	Inactive	Inactive	Inactive	Inactive
Calcitriol	1	1	Inactive	Inactive	Active	Inactive
Farnesol	5000	5	Inactive	Inactive	Inactive	Inactive
Lupenone	3660	5	Inactive	Inactive	Inactive	Inactive
Isopropenylcyclohexanol acetate	3200	5	Inactive	Inactive	Inactive	Inactive
4-Vinylguaiacol	1560	4	Inactive	Inactive	Inactive	Inactive
Limonene	4400	5	Inactive	Inactive	Inactive	Inactive

* Toxicity is classified as class 1–6. Class 1 is the most toxic and lethal, and class 6 is non-toxic.

## Data Availability

The original contributions presented in this study are included in the article and Supplementary Materials. Further inquiries can be directed to the corresponding authors.

## Supplementary Materials

The following supporting information can be downloaded at: https://www.mdpi.com/article/10.3390/ph18121901/s1, Figure S1, GC/MS analysis of the hexane extract; Figure S2, GC/MS analysis of the chloroform extract; Figure S3, GC/MS analysis of the ethyl acetate extract; Figure S4, HPLC chromatogram of the ethyl acetate extract; Figure S5, calibration curve for the determination of TPC. Table S1, bromatological analysis of the aerial parts of *E. hyemale* L.; Table S2, antibacterial activity of the hexane, chloroform, and ethyl acetate extracts from *E. hyemale* against Gram-positive and Gram-negative bacteria; Table S3. LC_50_ of the hexane, chloroform, and ethyl acetate extracts from *E. hyemale* L. against *C. elegans* nematodes.
