# Supplementary material for: Equisetum hyemale L. Extracts: Phytochemistry, Biological Performance, ADMET Profiling, and Toxicity Predictions
Source: Pharmaceuticals (Basel). 2025 Dec 17;18(12):1901. doi: 10.3390/ph18121901 (PMC12736147; doi:10.3390/ph18121901)
Supplement: Supplementary file 1 [file pharmaceuticals-18-01901-s001.zip › Supplementary material.pdf]

Supplementary Material

Equisetum hyemale L. Extracts: Phytochemistry, Biological Performance, ADMET Profiling, and Toxicity Predictions

Yulianna Minutti-Calva [1], Karen Schürenkämper-Carrillo [1], Edwin E. Reza-Zaldívar [2], Oscar E. Del Razo-Rodríguez [3], Ian Vitola [4], Jorge Manuel Silva-Jara [4], J. Daniel Lozada-Ramírez [1], Daniel A. Jacobo-Velázquez [5], Diego E. Navarro-López [6], Marco Chávez-Tinoco[8], Edgar R. López-Mena [7,*], Jorge L. Mejía-Méndez [6,*], and Eugenio Sánchez-Arreola [1,*]

[1] Departamento de Ciencias Químico-Biológicas, Universidad de las Américas Puebla, Ex Hacienda Sta. Catarina Mártir S/N, San Andrés Cholula 72810, Mexico; yulianna.minuttica@udlap.mx (Y.M.-C.); karen.schurenkamperco@udlap.mx (K.S.-C.); jose.lozada@udlap.mx (J.D.L.-R.).

[2] Tecnologico de Monterrey, Escuela de Medicina y Ciencias de la Salud, Ave. Ignacio Morones Prieto 3000, Monterrey 64710, Nuevo León, Mexico.; edwin.reza@tec.mx (E.E.R.-Z.).

[3] Instituto de Ciencias Agropecuarias, Universidad Autónoma del Estado de Hidalgo, Santiago Tulantepec de Guerrero Lugo 43775, Mexico; oscare@uaeh.edu.mx (O.E.D.R.-R.).

[4] Departamento de Farmacobiología, Universidad de Guadalajara, CUCEI, Blvd. Marcelino García Barragán 1421, Olímpica, Guadalajara 44430, Jalisco, Mexico; ian.castro8800@alumnos.udg.mx (I.V.); jorge.silva@academicos.udg.mx (J.M.S.-J).

[5] Tecnologico de Monterrey, Escuela de Ingeniería y Ciencias, Av. Eugenio Garza Sada 2501 Sur, Monterrey 64849 NL, Mexico; djacobov@tec.mx

[6] Tecnologico de Monterrey, Escuela de Ingeniería y Ciencias, Epigmenio González 500, San Pablo, Santiago de Querétaro 76130, Mexico; diegonl@tec.mx

[7] Tecnologico de Monterrey, Escuela de Ingeniería y Ciencias, Av. Gral. Ramón Corona No 2514, Colonia Nuevo México, Zapopan 45121, Mexico

[8] Departamento de Genética del Desarrollo y Fisiología Molecular, Instituto de Biotecnología (IBT), Universidad Nacional Autónoma de México (UNAM), Cuernavaca, Mexico; marco.antonio.chavez@ibt.unam.mx (M.C.-T.).

* Correspondence: edgarl@tec.mx (E.R.L.-M.); mejia.jorge@tec.mx (J.L.M.-M.); eugenio.sanchez@udlap.mx (E.S.-A.).

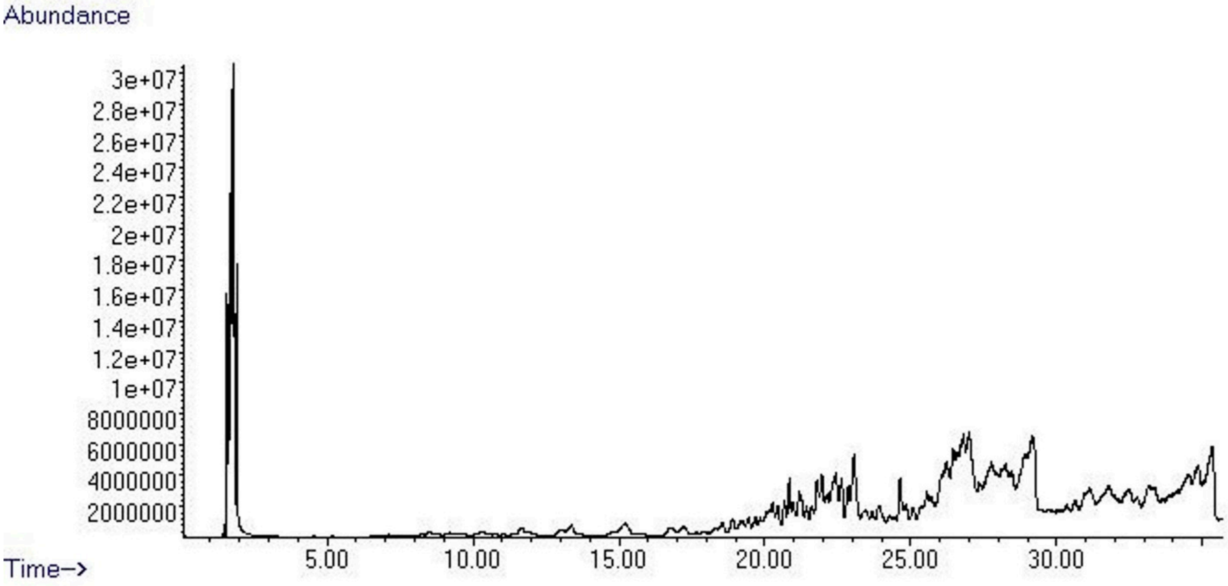

Figure S1. GC/MS analysis of the hexane extract.

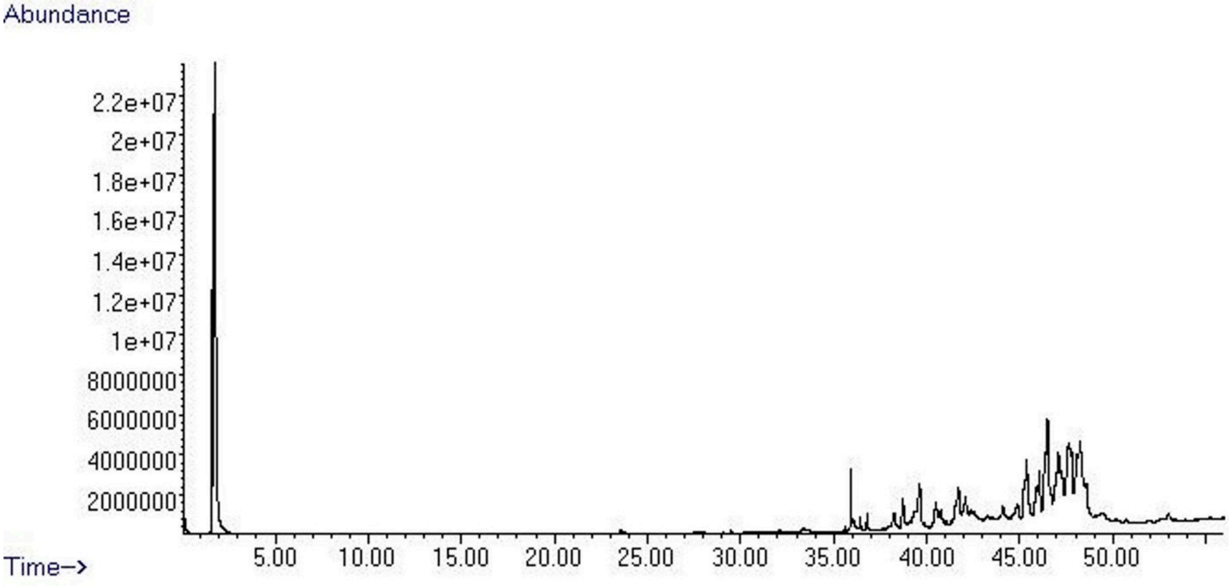

Figure S2. GC/MS analysis of the chloroform extract.

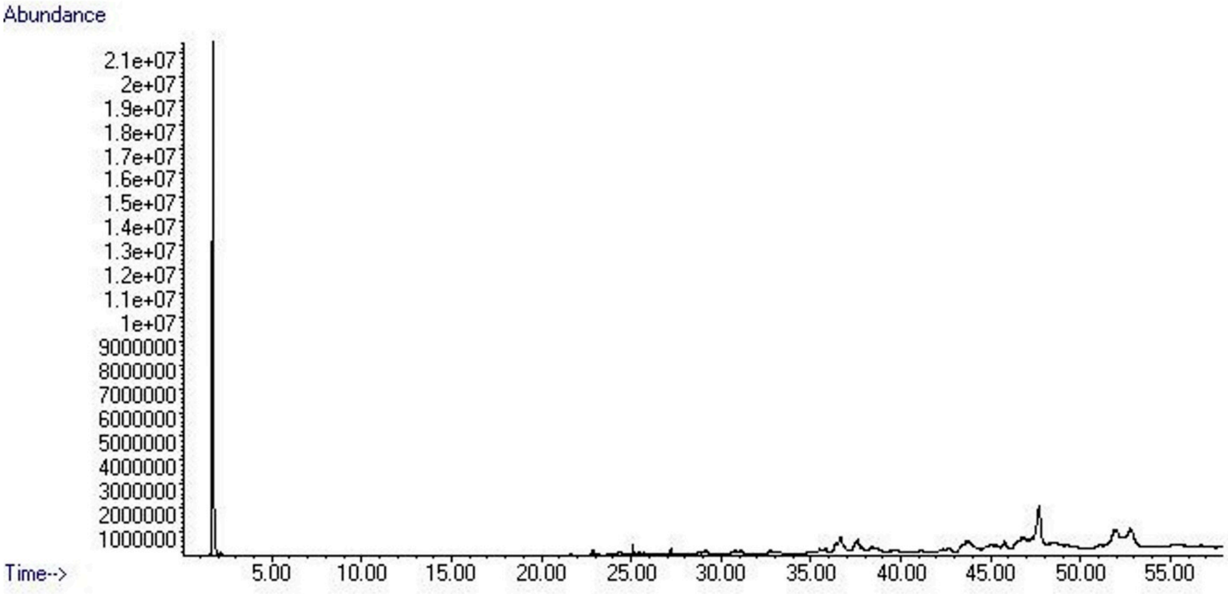

Figure S3. GC/MS analysis of the ethyl acetate extract.

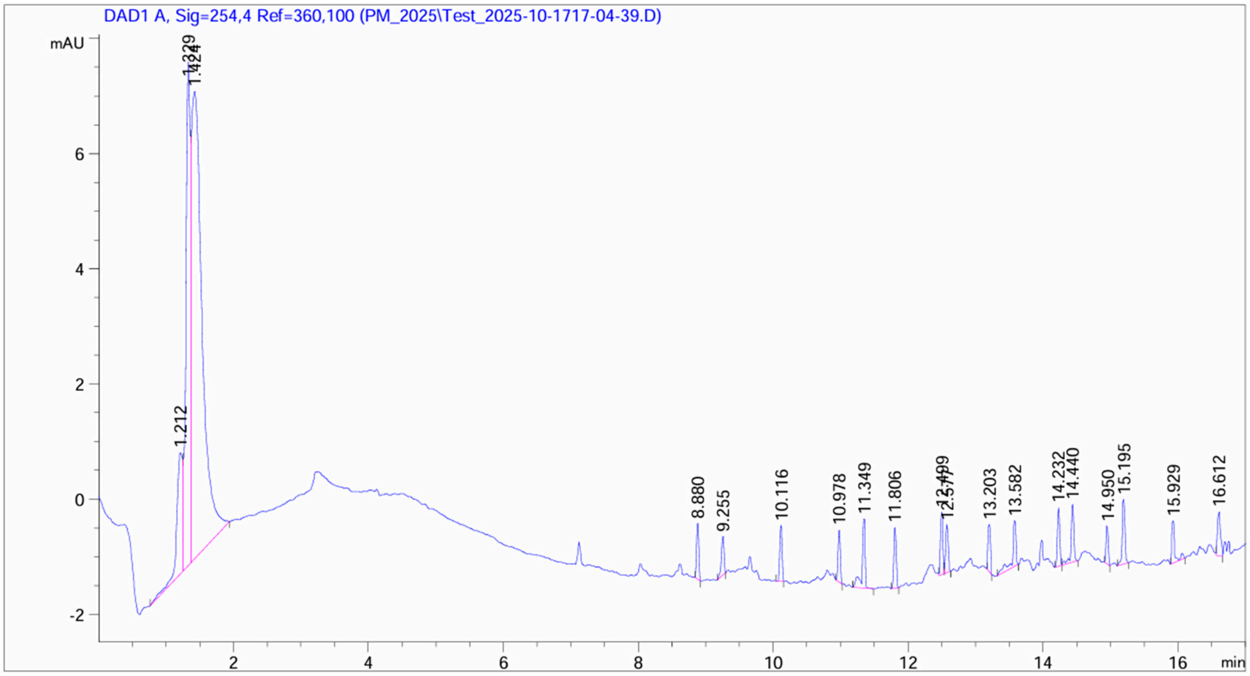

Figure S4. HPLC analysis of the ethyl acetate extract.

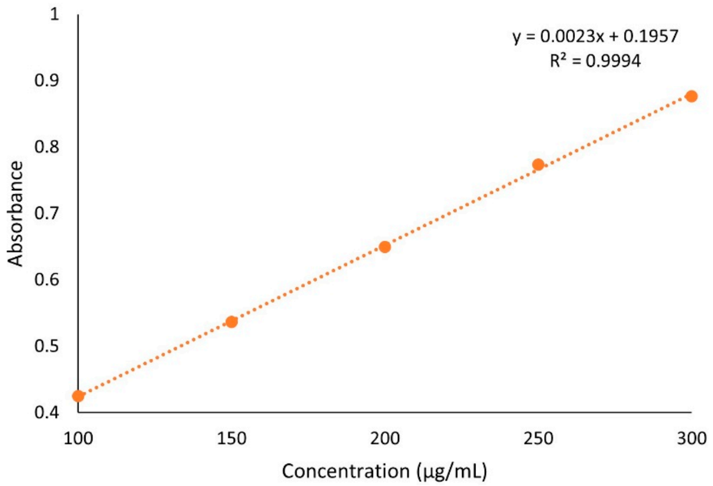

Figure S5. Calibration curve for the determination of TPC.

Table S1. Bromatological analysis of the aerial parts of *E. hyemale* L.

Variable	Content
Humidity	11.20
Ash	18.0
Crude protein	4.40
Crude fiber	38

Table S2. Antibacterial activity of the hexane, chloroform, and ethyl acetate extracts from *E. hyemale* L. against Gram-positive and Gram-negative bacteria.

Extract	SA	EC	KP	PA	PM
Hexane	ND	ND	ND	ND	ND
Chloroform	ND	ND	ND	ND	ND
Ethyl acetate	ND	ND	ND	ND	ND

Abbreviations: SA, *Staphylococcus aureus*; EC, *Escherichia coli*; KP, *Klebsiella pneumoniae*; PA, *Pseudomonas aeruginosa*; PM, *Proteus mirabilis*; ND, no detected.

Table S3. LC_{50} of the hexane, chloroform, and ethyl acetate extracts from *E. hyemale* L. against *C. elegans* nematodes. Concentrations are expressed in µg/mL.

Time (h)	H	Cl	EA
1	1184.31	12910	3263.18
2	229.15	258.52	237.23
3	180.30	196.36	190.66
4	152.53	159.52	160.83
5	142.10	142.57	146.98
6	130.94	130.36	129.33
